# Is there any electrophysiological evidence for subliminal error processing?

**DOI:** 10.3389/fnins.2013.00150

**Published:** 2013-08-29

**Authors:** Shani Shalgi, Leon Y. Deouell

**Affiliations:** ^1^Department of Cognitive Science, The Hebrew University of JerusalemJerusalem, Israel; ^2^Department of Psychology, Interdisciplinary Center for Neural Computation, and Edmond and Lily Safra Center for Brain Sciences, The Hebrew University of JerusalemJerusalem, Israel

**Keywords:** error awareness, Ne/ERN, confidence, subliminal processing, wagering

## Abstract

The role of error awareness in executive control and modification of behavior is not fully understood. In line with many recent studies showing that conscious awareness is unnecessary for numerous high-level processes such as strategic adjustments and decision making, it was suggested that error detection can also take place unconsciously. The Error Negativity (Ne) component, long established as a robust error-related component that differentiates between correct responses and errors, was a fine candidate to test this notion: if an Ne is elicited also by errors which are not consciously detected, it would imply a **subliminal process** involved in error monitoring that does not necessarily lead to conscious awareness of the error. Indeed, for the past decade, the repeated finding of a similar Ne for errors which became aware and errors that did not achieve awareness, compared to the smaller negativity elicited by correct responses (Correct Response Negativity; CRN), has lent the Ne the prestigious status of an index of subliminal error processing. However, there were several notable exceptions to these findings. The study in the focus of this review (Shalgi and Deouell, 2012) sheds new light on both types of previous results. We found that error detection as reflected by the Ne is correlated with subjective awareness: when awareness (or more importantly lack thereof) is more strictly determined using the wagering paradigm, no Ne is elicited without awareness. This result effectively resolves the issue of why there are many conflicting findings regarding the Ne and error awareness. The average Ne amplitude appears to be influenced by individual criteria for error reporting and therefore, studies containing different mixtures of participants who are more confident of their own performance or less confident, or paradigms that either encourage or don't encourage reporting low confidence errors will show different results. Based on this evidence, it is no longer possible to unquestioningly uphold the notion that the amplitude of the Ne is unrelated to subjective awareness, and therefore, that errors are detected without conscious awareness.

## Introduction

The human brain is wondrously efficient and complex, but by no means perfect, and errors are ubiquitous. Sometimes, we are acutely aware of having made an error, leading to the “oops” sensation, whereas at other times, errors go unnoticed. The detection of errors is crucial for successfully navigating through life and effectively performing tasks. Error detection affords us the opportunity to modify behavior, which is essential for learning. Error detection may be particularly critical in high-risk tasks such as operating machinery or driving a car, where immediate error detection and correction can be vital to survival. What is not clear is the extent to which error detection occurs as a conscious or an unconscious process. For example, is conscious error detection a precondition for error correction or strategic adjustment of future behavior? Reduced awareness of errors has in fact been associated with a number of clinical conditions (ADHD, O'Connell et al., 2009; psychopathy, Brazil et al., 2009; drug addiction, Hester et al., 2009). Therefore, a more thorough understanding of the conditions under which errors reach consciousness and the neural correlates of **error awareness** may also have a significant clinical value.

The question of how consciousness is involved in **error processing** is interesting also because it is part of a much larger and profound question about the role of conscious awareness in our behavior. What function does consciousness serve? One way to answer this question is by elimination, that is, to ask what type of operations can be carried out in the absence of awareness. While it is generally accepted today that several perceptual, emotional and cognitive processes can run outside of awareness (e.g., Pessoa, 2005; Pessiglione et al., 2007, 2008; Bargh and Morsella, 2008; Sklar et al., 2012), it is also a common assumption that executive control operations do require conscious awareness for optimal performance (Mandler, 2002; Koch, 2004; Baars, 2005). This makes intuitive sense, as we usually become aware of those aspects in the internal or external environment that interfere with or interrupt routine action, or that defy our expectations and predictions, and errors, especially in simple tasks, are paradigmatically unexpected. Nevertheless, recent studies suggest that even cognitive control processes can be initiated unconsciously in some circumstances (see van Gaal and Lamme, 2012; van Gaal et al., 2012; Hassin, 2013 for reviews on this topic). Thus, the extent to which higher-level cognitive functions such as error processing and strategic adjustment of behavior can occur subconsciously remains unclear.

Throughout this review, we differentiate between two types of errors: Mistakes and Slips (see Reason, 1990). Slips are defined as errors that occur when the information needed to make the correct response is available to the decision making process (i.e., stimulus processing can be completed given sufficient time, and the task is understood). In contrast, Mistakes are errors made when the information or the algorithm needed to make the correct response is insufficient. For example, a typographical error of a familiar word is a Slip, while a wrong guess at the spelling of a novel word, or in solving a complex math problem, is a Mistake. In laboratory tasks, a Slip error could occur due to fast responding when the stimuli was seen but before it has been completely processed, or due to momentary lapse of attention to the task, and a Mistake could occur when the participant blinks at the time of the stimulus or did not fully understand the task. This implies that every Slip error can potentially be detected, whereas the same missing information that caused the Mistake would also hinder its detection. In our discussion of error processing in the brain, we refer only to Slip type errors, as processing can occur only in the presence of some information regarding the correct response^1^.

## Electrophysiological indices of error processing

The scientific study of error monitoring began during the 1960s with a series of psychophysics studies. Rabbitt and co-workers' pioneering work established that error monitoring relies on post-decision processing (e.g., Rabbitt, 1966, 2002; Rabbitt and Vyas, 1981). The in-depth study of the physiological characteristics of error processing began only two decades ago, with the discovery of two response-locked **event related potentials (ERPs)** that are enhanced following errors in contrast to correct responses. The first of these, termed the Error Negativity (Ne, Falkenstein et al., 1991; or ERN, Error-Related Negativity, Gehring et al., 1993) is a negative deflection in the ERP with a fronto-central distribution that begins around the time of the incorrect response^2^, and peaks roughly 50–100 ms thereafter. At the time of the Ne, ERPs locked to correct responses typically show a significantly smaller negativity termed the Correct-Related Negativity (CRN; Ford, 1999; Vidal et al., 2000). It has recently been suggested using Independent Component Analysis (ICA) and source localization that the Ne and the CRN reflect the same underlying brain activity (Roger et al., 2010). The second error-related component is termed the Error Positivity (Pe, Falkenstein et al., 1991). The Pe is a more sustained component, which is absent after correct responses and is maximal at centro-parietal electrodes, peaking between 300 and 500 ms after the error. Both components appear independently of stimulus or response modality (Van `t Ent and Apkarian, 1999; Falkenstein et al., 2000; Shalgi et al., 2009) and across a number of paradigms designed to elicit errors, such as response inhibition tasks and choice reaction-time tasks. Based on converging evidence from different research methods, both components were localized to the Anterior Cingulate Cortex (ACC), albeit in different regions. The Ne is thought to originate from the dorsal/caudal ACC (ERP dipole localization: Dehaene et al., 1994; van Boxtel et al., 2005; O'Connell et al., 2007; LORETA: Herrmann et al., 2004; Trial-by-trial couplings of EEG and fMRI signals: Debener et al., 2005; Intracerebral recordings: Brazdil et al., 2002; though see Agam et al., 2011, for a more posterior location within the ACC). The Pe was localized to a comparatively more rostral region (van Veen and Carter, 2002; van Boxtel et al., 2005 and O'Connell et al., 2007, who also found contributions from the precuneus). Although some recent studies have questioned a direct relationship between the ACC and the Ne (see Orr and Hester, 2012), as a paralimbic area the ACC is uniquely positioned to integrate salient cognitive and emotional information with motor responses. It is active during the performance of complicated tasks, especially those involving conflict, and is thought to play a prominent role in the executive control of cognition (Botvinick et al., 2004; Ridderinkhof et al., 2004). The Ne and Pe also dissociate in their sensitivity to experimental manipulations, pharmacological substances and personality traits (for an overview, see Overbeek et al., 2005) and are therefore thought to play different roles in error processing. Due to its early latency after the error, the Ne has been linked to automatic, pre-conscious error monitoring, while the Pe has usually been associated with later conscious processes such as compensatory adjustments, emotional appraisal and mainly error awareness. However, the exact roles of both the Ne and Pe are still a matter of debate. This review will focus solely on the role of the Ne with regard to error awareness.

## Error awareness and the Ne

The prevailing dogma for the past decade has been that the errors we make are registered by our brain even if we are unaware of them. Nieuwenhuis et al. (2001) showed that the Ne was equally seen after errors which subjects reported (henceforth “Aware Errors”), and after errors that were unnoticed (henceforth “Unaware Errors”). This result was striking because it implied that the whole complex and sophisticated sequence of processing—from stimulus recognition, decision making, up to the level of response selection and response monitoring—can be completed “below the surface.” That is, not only are stimuli and tasks processed unconsciously up to the semantic level (e.g., Costello et al., 2009; Mudrik et al., 2011; for a review see Kouider and Dehaene, 2007), but the brain also knows at a very early stage (perhaps even before the response was actually made) what should have been done regarding the stimuli and what was actually done. However, for some reason this information is not always relayed to consciousness. This starkly contradicts one of the most popular theories of the function of conscious deliberation, which is to deal with unexpected, non-routine situations (of which the error scenario is paradigmatic), designing intentional strategies for their handling, modifying behavior, and learning for future occasions. Although a prior study linked the Ne amplitude to subjective confidence of the response (Scheffers and Coles, 2000), the finding of equal amplitude Ne for aware and unaware errors was replicated several times in eleven different studies, including in our hands (Endrass et al., 2005, 2007, 2012; O'Connell et al., 2007, 2009; Pavone et al., 2009; Shalgi et al., 2009; Shalgi and Deouell, 2010; Dhar et al., 2011; Hughes and Yeung, 2011; for a review see Wessel, 2012), and led to the pervasive notion that error monitoring, as reflected by the Ne, can occur regardless of conscious awareness (e.g., Simons, 2009). The findings of these ERP studies were further supported by two event-related fMRI studies, which showed that activation of the ACC region associated with the generation of the Ne did not differ between Aware and Unaware Errors, while Aware Errors were associated with larger bilateral activation of prefrontal and parietal regions (Hester et al., 2005), or left anterior insula activity (Klein et al., 2007) compared to Unaware Errors. However, as more evidence accumulated, it became harder to ignore the data showing that error processing might not occur without one being aware of the error. Six ERP studies (Scheffers and Coles, 2000; Maier et al., 2008; Steinhauser and Yeung, 2010; Woodman, 2010; Hewig et al., 2011; Wessel et al., 2011) showed that the Ne was significantly more negative for Aware Errors than Unaware Errors and correct responses, and three more studies which did not directly compare Aware and Unaware Errors were consistent with this notion (Praamstra et al., 2003; Pailing and Segalowitz, 2004; Selimbeyoglu et al., 2012).

A simple explanation which would reconcile the difference between the results of the two groups of studies might be that in those studies which found no difference between the Aware and Unaware Ne, many subjectively Aware Errors were not reported. In such cases, the group of trials designated “Unaware Errors” would be “contaminated” by many Aware errors with large Ne's (see also Wessel, 2012). This would result in a reduction of the difference between the Aware and the Unaware Error ERPs (which are the average of trials in each group), becoming statistically insignificant. In fact, five of the studies which found no effect of awareness on the Ne, reported that its amplitude was numerically larger for Aware compared to Unaware Errors (Endrass et al., 2007; Shalgi et al., 2009; Shalgi and Deouell, 2010; Dhar et al., 2011; Hughes and Yeung, 2011; but see O'Connell et al., 2007, for the opposite result). However, there is no systematical difference between the experimental design of the experiments that did and did not find an effect of awareness on the Ne that would cause a response bias toward not signaling an error (see Wessel et al., 2011). In addition, error awareness rates in the different groups of studies were not noticeably different. Thus, the puzzle remained—can the Ne be taken as a signature of error detection which is independent of awareness of the error?

In his recent review of the relationship of the Ne with error awareness, which summarized thirteen studies, Wessel (2012) ruled out two more hypotheses as possible explanations for the presence or absence of error awareness effects on the Ne amplitude in different studies: (1) whether the stimuli were degraded or not in the given studies, and (2) whether errors were corrected or not (following Steinhauser and Yeung, 2010, who proposed that the amplitude of the Ne is an index of fast and automatic error correction). As an alternative, Wessel (2012) suggested that the studies that did not find a significant difference between the Ne for Aware and Unaware Errors but nevertheless, showed a numerical difference, may have suffered from low statistical power, increasing the probability of a type-2 error, i.e., the probability of not rejecting a null hypothesis, even though the alternative hypothesis is true. In light of this suggestion, Orr and Hester (2012) re-analyzed the fMRI data of three studies of error awareness, forming a composite sample of 56 subjects to increase statistical power. In contrast to their initial study (Hester et al., 2005, whose participants were included in this re-analysis), Orr and Hester found that the error-related dorsal ACC activity was significantly greater during Aware Errors compared to Unaware Errors, supporting the possibility that their initial null result was due to low statistical power. However, there are two important reasons to question the ability of the low-power hypothesis to adequately explain all the studies that failed to find an awareness effect on the Ne. First, numerical differences between Aware and Unaware Errors were found only in a few of the studies (in fact O'Connell et al., 2007, found a non-significant difference in the opposite direction). Second and most importantly, the finding of a similar Ne for Aware and Unaware errors cannot be viewed simply as a null result, because the imperative finding in those studies is in fact that Unaware Errors elicit a significant error-related component compared to correct responses (see Dhar et al., 2011, for an exception, and Endrass et al., 2005, who did not measure the Ne of correct responses).

**Figure 1:**
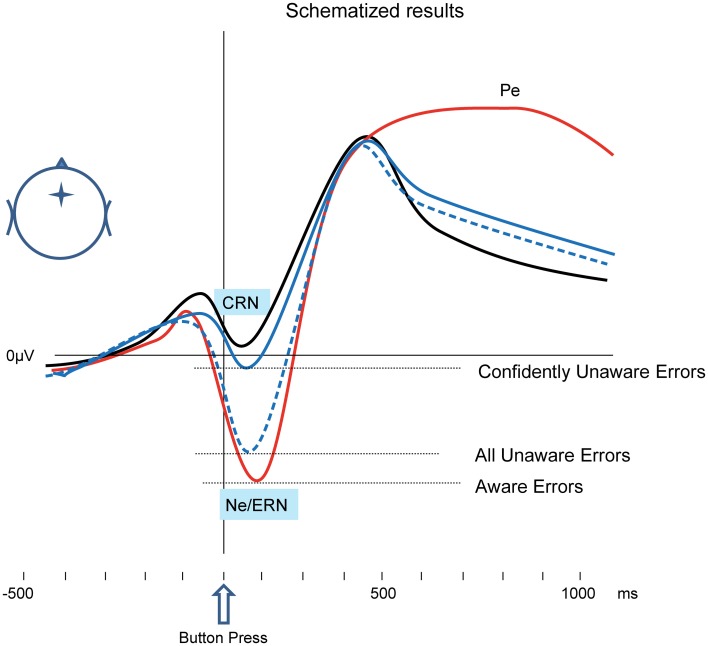
**Schematized results, based on Shalgi et al. (2009); Shalgi and Deouell (2012).** In typical Ne studies, nominally Unaware Errors may elicit an Ne-like response (broken blue line), which is similar or a little smaller than the Ne elicited by Aware Errors (red line). However, when only errors which were confidently missed (a high bet on correct) are considered, the response to Unaware Errors is similar to the CRN elicited by correct responses (black line).

The study in the focus of the current review (Shalgi and Deouell, 2012) proposed and empirically tested a solution to the above debate, which explains both types of results (difference/no difference between the Ne of Aware and Unaware Errors). In a choice-reaction time task, we asked our participants, on every trial, to judge whether they were accurate in their response or not (accuracy judgment). Immediately following this decision, we asked participants to bet money on their just-made accuracy judgment (**wagering technique**; Ullsperger et al., 2010). By examining the amount of their bet we effectively gained a measure of their subjective confidence in whether they were right or wrong in their accuracy judgment. We found that when participants made an error, *yet were certain that they had not erred* (indicated by their willingness to bet high that they had made a correct response), the Ne was significantly smaller than when subjects made an error, reported it, and were certain of it (betting high that they had indeed made an error). Moreover, when subjects erred and were certain that they did not, the Ne was comparable to the CRN elicited by correct responses of which they were confident. Conversely, when subjects were correct but were uncertain of their performance (betting low on whether they were correct) or when they reported they erred but were uncertain of it, an intermediate CRN/Ne was elicited by all types of responses (Correct, Aware Errors, and Unaware Errors). These findings (schematized in Figure 1) suggest that the error detection as reflected by the Ne *is* after all dependent on subjective awareness of the error—when the level of awareness is more strictly determined using the wagering paradigm^3^, no Ne is elicited without awareness. Thus, the Ne cannot be taken as evidence for error detection which is independent of error awareness.

**Figure 2:**
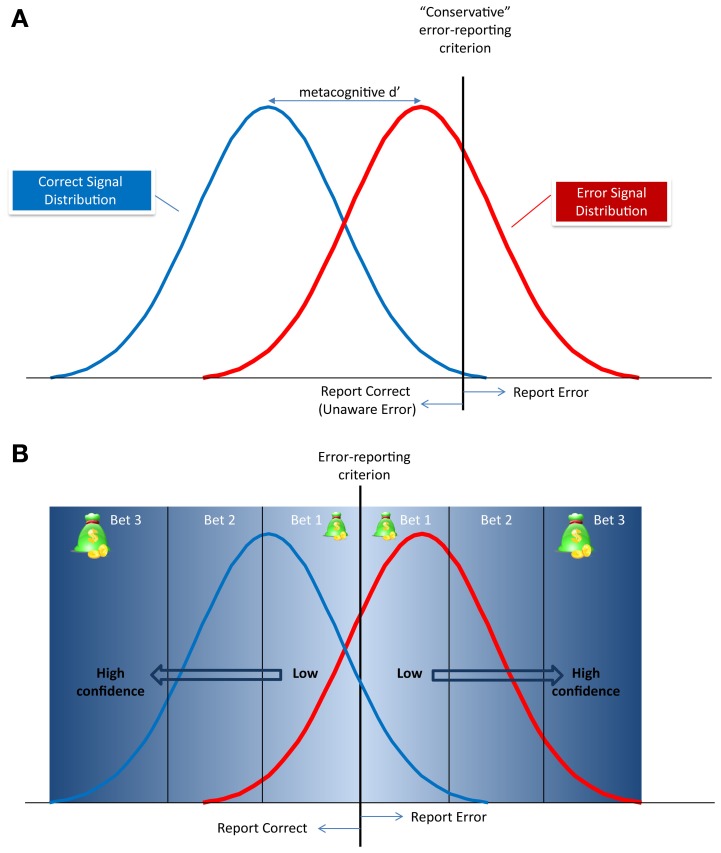
**Metacognitive signal detection model of error reporting. (A)** The correct and error signal probability distributions and the decision criteria for reporting an error (vertical dotted line). The distance between the peaks of the distributions is the metacognitive d' and defines the discriminability of an error from a correct response. In this example, the criterion is conservative, meaning that the subject will report only errors whose signal has very little overlap with the correct response signal. All other errors will be reported as “correct” as therefore, be classified as Unaware Errors. **(B)** The relationship between the metacognitive criterion and the subject's confidence, or betting scheme. The further the signal is from the criterion, the higher the confidence in the metacognitive decision, and the higher subjects will be willing to bet.

Under the framework of **signal detection theory** (SDT; Green and Swets, 1966), both the “signal” (in our case, the error signal) and the background “noise” are associated with neuronal activity patterns that have a probabilistic distribution, and these distributions may overlap. This overlap creates ambiguous situations, requiring the subjects to make a decision (in the present case, to indicate that an error was or wasn't made), even though the evidence is compatible with both options. SDT suggests that subjects set a threshold (criterion), and decide in favor of a “signal” only if the evidence exceeds this criterion. Under this framework, if subjects are conservative, they may refrain from declaring an error, even though there is a considerable evidence in favor of one (Figure 2A). Where does the wagering technique come into play within this scenario? Even if human subjects' overt responses are based on their personal criterion (which is presumed to be stable within an experiment), they nonetheless make individual responses with different degrees of confidence. The process of confidence judgment is still debated (Clarke, 1959; Kunimoto et al., 2001; Pleskac and Busemeyer, 2010; Maniscalco and Lau, 2012), and the specific arguments are beyond the scope of this review. However, consistent with models of SDT addressing metacognitive judgments such as error detection (Clarke, 1959; Maniscalco and Lau, 2012; Rahnev et al., 2012), it seems reasonable to assume that, for a given subject with a set discrimination criterion, decisions made with high confidence occur when ambiguity is minimal. That is, when the signal in a given trial is far from the decision criterion (Figure 2B), or when the discriminability between the error and no error condition is greater (larger metacognitive d')^4^. Note that here the confidence measured is about the secondary decision (also known as the “Type 2 decision,” see Galvin et al., 2003) about the error signal (“did I make and error or not”), and not about the initial decision about the stimulus (“Type 1 decision”). Thus, by examining only trials with high bets, one can limit the analysis to trials where the subjects were fully aware of the error, or, more importantly, truly had no awareness of the error.

The results thus effectively resolve the issue of why there are conflicting findings regarding the Ne and error awareness, suggesting that there may be a range of subjective experiences of making an error, rather than an all-or-none phenomenon. The Ne amplitude thus appears to be influenced by the level of discriminability (in signal detection terms) as well as the criteria different individuals have for error reporting; studies containing different mixtures of participants who are more or less conservative in their error reporting will yield different results. Additionally, some paradigms may either encourage subjects to report errors even when they are less confident, while others may discourage reporting low-confidence errors, also affecting the results. Specifically, if something in the design of the experiment, the task instructions, or an experimenter bias, encourages the subjects to report errors only when they are highly confident, or if because of random selection the subjects in the experiment are relatively conservative, and no confidence measures are used, many error trials classified as Unaware Errors will in fact have some level of awareness, thus increasing the amplitude of the Ne in the putatively “unaware” category.

Recently, a study by Steinhauser and Yeung (2010) directly examined the effect of manipulation of the error reporting criteria on the amplitude of the Ne. The authors used a brightness discrimination task and asked subjects to judge whether they were right or wrong in every trial. They used two different incentives to encourage participants to adopt either a high (report an error only if you are very sure) or a low criterion for reporting their errors. Steinhauser and Yeung's main results were consistent with ours: when the high criterion was imposed, the Ne was significantly larger for Aware Errors than Unaware Errors. Inconsistent with this (as well as with our above predictions) there was no difference between the Ne elicited by Aware Errors under the low and high criterion conditions. It is hard to reconcile these two results, and further experiments will be needed to reveal whether the type of task (threshold discrimination of brightness vs. discrimination of highly visible objects) is critical in determining the effect of criterion on the Ne, or whether the direct manipulation of error signaling criterion (“Type 2 decision”) is indeed equivalent to manipulating the subjective level of confidence and awareness of the error. Putatively, once a Type 2 criterion is set and an error signal is elicited, if this criterion is crossed, confidence in this Type 2 decision (a type of 3rd level decision) may still be as variable, regardless of the initial criterion.

## The functional significance of the Ne

Does the sensitivity of the Ne to subjective error awareness contribute to the debate about its functional significance? Three major models have been suggested to explain the Ne: (1) Error Detection (Falkenstein et al., 1991; Coles et al., 2001), (2) Reinforcement Learning (Holroyd and Coles, 2002) and (3) Conflict Detection (Botvinick et al., 2004; Yeung et al., 2004). According to the Error Detection account, a dedicated module for detecting errors exists in the brain. This module detects the mismatch between the actual response committed and the intended response, and the Ne is seen to reflect the size of this mismatch. The Reinforcement Learning theory also suggests a generic error processing system, comprised of a comparator and of a remedial action system. Upon detecting an error, the comparator, located at a subcortical level (the basal ganglia) transmits a learning signal via the mesencephalic dopaminergic system to the remedial action system situated in the ACC, indicating that the outcome of the current action was worse than expected (negative reward). The Ne is generated by the arrival of the error signal at the remedial action system. According to this account, the ACC does not monitor errors *per se*, but uses the learning signal to adapt the response selection process^5^. According to the Conflict Detection account, the Ne reflects the amount of response conflict present after an error has been committed. The post-error response conflict is a consequence of continued processing of the stimulus that leads to post-error activation of the correct response and therefore, conflict with the incorrect response just produced^6^. The presence of response conflict indicates situations in which errors are likely to occur and hence require attention. Our finding of a larger Ne for confident errors seems to be compatible with both the Error-Detection and the Reinforcement Learning accounts. When an error is certain, there should be a large mismatch between the actual response and the required response compared to an uncertain error (hence larger error-detection signal), and indeed the Ne amplitude is larger for certain than uncertain errors. Concomitantly, evident errors are probably considered worse outcomes (i.e., negative reward) than unsure errors, requiring readjustments (hence learning). Are the results also consistent with the Conflict Detection account? Scheffers and Coles (2000) argued that Ne variation with subjective confidence argues against this model. They reasoned that the largest conflict should occur when subjects were uncertain of their primary response, whereas in fact, in their hands and ours, uncertainty yields smaller Ne. However, the relationship between confidence and conflict is not straightforward in our view. The response (associated with the Ne/CRN) and the confidence judgment are dissociated in time, and both stimulus and response processing continue to evolve between the primary response and the accuracy judgment. In one scenario, a large conflict at the time of the response may lead to uncertainty regarding the response at the time the accuracy judgment is given. In another scenario, the same conflict may have been fully resolved by the time of the accuracy judgment, leading to high certainty. Thus, confidence in accuracy judgments does not correspond in a straightforward manner to high or low conflict at the time of the response. Considering only the confident responses however, it is conceivable that a higher conflict occurred in cases of Aware Errors (if both the correct and incorrect responses were activated) than in the case of Unaware Errors or Correct responses, in which one response, either the correct or the incorrect one, dominated. Therefore, in our view, the findings of our recent experiment (Shalgi and Deouell, 2012) and those of Scheffers and Coles (2000) do not unequivocally distinguish between the Error Detection, Reinforcement Learning, and the Conflict Detection hypotheses of the Ne. They do however, challenge the notion of error detection without awareness.

## Unconscious error processing?

The latency of the Ne, immediately or even at the same time as the commission of an error, suggests that it must be based on quickly available feed-forward information and not require other inputs such as proprioceptive or sensory feedback (Allain et al., 2004; de Bruijn et al., 2004; Mathalon et al., 2004). The early latency is what led to the initial proposal that the Ne was an index of automatic, unaware error processing. This is because conscious awareness is thought to be a slower process (Rabbitt, 2002), which comes about only after the accumulating evidence about erroneous behavior, based on numerous sources of information, exceeds some threshold and activates sufficiently large neural networks (Dehaene et al., 2006). In this respect, the variation of the Ne with the level of awareness of the error is surprising. We propose (in line with Wessel, 2012) that the initial error signal indexed by the Ne does not reflect error awareness *per se*, but rather that it is a prerequisite of this process, and thus, correlated with it. Possibly, if some Ne is generated, a second, compound internal error signal based on other sources of information (including later events, such as proprioceptive feedback, autonomic responses and sensory input) may exceed the threshold for error awareness.

**Figure 3:**
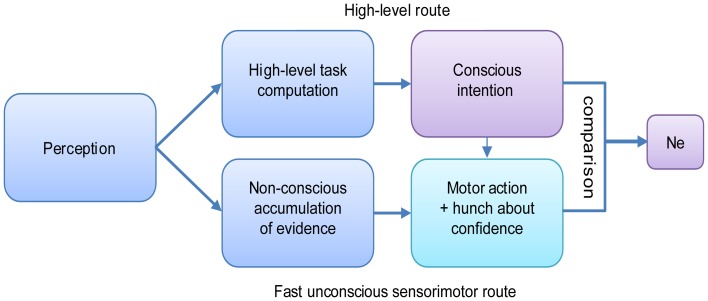
**The dual-route model of decision making, based on Del Cul et al. (2009) as expanded by Charles et al. (2013)**.

Thus, as opposed to prevailing views, the Ne seems to be correlated with, and possibly a prerequisite to, awareness of an error. Does this mean that without an Ne one cannot be aware of an error? A very recent study by Charles et al. (2013) suggests some types of awareness can occur without eliciting an Ne. Their results showed above-chance error awareness, detected using a forced choice technique, to stimuli that were effectively masked. For these correctly detected (in fact guessed) errors, no Ne was recorded. This finding was interpreted in light of an extension of the dual-route model of decision-making proposed by Del Cul et al. (2009; Figure 3). According to the dual-route model, two parallel routes, with different noise levels and thresholds, accumulate sensory evidence toward a categorical decision on the same input stimulus. One is a fast, non-conscious sensorimotor route (bottom row, Figure 3), and one is a slower conscious decision route (high-level route, top row). A motor response is emitted by the route that first reaches its decision threshold. Usually, we try to guide our actions by our conscious intention, but sometimes our actions start earlier through the fast route, and then we might slip and make an error. According to Charles et al. (2013), the Ne is generated as a result of a discrepancy between the responses computed by the two routes (the difference between intended and executed action).

By this model, the Ne is generated only when a conscious intention exists, i.e., when the conscious route has crossed its threshold. If no conscious intention is generated (e.g., when the stimulus is below threshold), no comparison can be initiated, yet a “hunch,” generated based on rudimentary (unconscious) evaluation of evidence, might be used later for the above chance guessing. Thus, the model can explain both the correlation between error awareness and the Ne when the stimulus is consciously perceived, and attenuated above-chance error awareness without an Ne when the stimulus is not consciously perceived and forced-choice error signaling is used.

## Methodological implications

Our findings underscore the importance of careful evaluation when it comes to the treacherous issue of conscious awareness vs. subliminal processing Simply asking for subjective report is notoriously unreliable. In signal detection theory terms, it confounds discriminability (of an error in our case) and the criterion for reporting. The wagering process we used does not solve the problem altogether, but it provides a way for determining the distance from the criterion: reluctance to bet high suggests a decision close to the criterion, and so the determination of awareness in these trials is murky. In contrast, willingness to bet high suggests the decision was made well above the criterion, making the exact position of the criterion less critical.

The addition of additional measures, like wagering or the forced choice method, is often cumbersome and disrupts the flow of the task even more than simple accuracy judgments. Therefore, an important next step in the study of the Ne as an index of subjective error processing is to identify those factors that influence overall error reporting (e.g., task difficulty, stimulus ambiguity), and the individual differences (e.g., traits) that lead to variable error reporting.

## Conclusion

Error awareness is a fascinating topic. It underlies numerous philosophical and cognitive-psychological questions such as the possibility of acting without awareness (the “zombie” mode; Koch and Crick, 2001) and the meaning of awareness in executive function. In Shalgi and Deouell (2012), we presented evidence that the error-related component previously taken as support for the existence of subliminal error processing (the Ne) is actually modulated by subjective confidence that an error has occurred, and in fact correlates with conscious awareness of the error. This leaves us with no clear-cut electrophysiological evidence for the unconscious processing of errors.

### Conflict of interest statement

The authors declare that the research was conducted in the absence of any commercial or financial relationships that could be construed as a potential conflict of interest.

## Footnotes

^1^Slips may go undetected because of failures to note the correct answer for a given trial, or because of failures to note a subtle erroneous response made (e.g., when the eye moves slightly in the wrong direction in an anti-saccade task, before a corrective movement is executed). A special type of errors that do not reach consciousness but are implicitly corrected, such as movement errors corrected by the cerebellum during sensory-motor adaptation will not be discussed here.

^2^While the Ne usually begins before the actual response time measured from a button press, it probably is more closely locked to the incorrect electromyographic (EMG) signal onset, which is not always measured in EEG experiments.

^3^While the wagering technique has become popular in the field of consciousness, it has also been criticized (e.g., Clifford et al., 2008; Fleming and Dolan, 2010; Sandberg et al., 2010). Wagering related factors such as loss/risk aversion or subjective monetary strategies were brought up mainly to question the use of low bets as a signature of low confidence. However, in our use of wagering these concerns only serve to bolster the use of the high bets as a relatively pure measure of high confidence (for example, if subjects sometimes refrained from betting high due to loss aversion, then trials with the highest bet would undoubtedly be trials in which confidence in the accuracy judgment was strong enough to overcome loss aversion). Moreover, we did not directly compare between low and high bets.

^4^Indeed, our subjects varied considerably in their wagering performance; some subjects bet mainly using the highest bet and others mainly on the lowest amount, suggesting different d' for detecting errors.

^5^A recent study suggests that whenever an outcome of an action violates expectancies, not necessarily related to reward, the ACC appears to be engaged in evaluating the necessity of behavioral adaptation (Wessel et al., 2012). Using both EEG and fMRI, Wessel and colleagues established that the Ne and the novelty N2 which indexes processing of generally infrequent or surprising stimuli share a common neuroanatomical substrate in the ACC. The authors thus suggest generalizing the Reinforcement Learning theory of the Ne beyond reward expectancies. However, as the definition of reward is highly flexible, one can argue that novelty or surprise are rewarding (either positively or negatively) and remain with the original theory.

^6^Note that response conflict is different from perceptual conflict. There are cases where the perceptual conflict is large but the response conflict is small (e.g., Danielmeier et al., 2009).

## Key Concept

Subliminal processing: Many, if not most, of our mental processes are inaccessible to conscious introspection, that is, we have no subjective experience of them. These subliminal processes, which are not available for explicit reporting, can be manifested in behavior (for example in post-error slowing) or by neuroimaging techniques (for example event-related potentials).
Error processing: The study of how an error is processed by the brain, under the assumption that errors are processed differentially than correct responses. It is debated whether errors are specifically registered as incorrect responses or are simply events linked to a large response conflict.
Error awareness: The study of how an error becomes recognized consciously. Unanswered questions in the field of error processing are: What brings about awareness of certain errors but not of others? Are errors which we are unaware of making, subliminally processed by the brain as errors at all? Or are they simply considered correct responses and not processed differentially?
Event Related Potentials (ERPs): The electrophysiological neural response measured on the scalp which is directly related to a specific sensory, cognitive, or motor event (such as a certain stimulus or a response to a stimulus). Each ERP has a specific scalp distribution, latency and polarity (positive/negative) which defines it. ERPs are extracted by time-locking the raw EEG measurement to the triggering event, and usually averaging over several repetitions of the same event in order to increase the signal-to-noise ratio. Ideally, ERP components serve as physiological indices of specific cognitive functions.
Wagering technique: A key problem of experiments that investigate conscious and subliminal processing is to objectively separate those stimuli or events which participants are consciously aware of and those which they are unaware of, due to the subjective nature of consciousness. The wagering technique is a method which has been proposed to solve this problem. In every trial of an experiment, after the response to primary task, the participant is asked to bet some money on whether their response is correct. If they are correct, they win this money; otherwise, they lose it. The basic idea is that people who know that they have information are usually willing to bet on it; that is, they are willing to “put their money where their mouth is.” In terms of assessing error awareness, the participants are first asked to judge whether their primary response was correct or incorrect, and then asked to bet on that judgment. In trials in which the participant made an error and judges it as correct, a high bet indicates that the participant was indeed unaware of their erroneous response, whereas a low bet would indicate some awareness of the error (of a trial that would otherwise be classified as an unnoticed error). The wagering method, by exploiting people's desire to make money, is a more objective measure of awareness than asking participants to explicitly report on their confidence.
Signal detection theory: Borrowing from the field of communications, SDT models our ability to discern between information-bearing patterns (the signal) and irrelevant patterns (noise). In the present case of error detection, the signal would be an error, and the “noise” refers to correct responses. Under the assumption that the noise signal has some probability distribution of possible patterns and the signal has another distribution, an observer needs to make a decision for every pattern, deciding whether it belongs to the noise distribution or to the signal distribution. Ambiguity arises when some patterns may come from either distribution, requiring a decision criterion (or threshold). The criterion specifies for which patterns the system will make a “signal” or “no signal” response, and depends on both the physical properties of the signal and the psychological state of the individual (e.g., experience, expectations, attention). For example, if a subject has a conservative response criterion for reporting errors, they may decide to report one only when they are absolutely sure that an error has been made (that is, when there is no ambiguity) and not report an error in any other case. Conversely, if one has a liberal criterion, they may report an error any time they feel their performance was less than perfect (that is, for all cases of ambiguity). Within this model, confidence may be determined by the distance from the decision threshold; the further away one is from the one's threshold, the more confident is the decision.
